# Effect of high-frequency repetitive transcranial magnetic stimulation under different intensities upon rehabilitation of chronic pelvic pain syndrome: protocol for a randomized controlled trial

**DOI:** 10.1186/s13063-023-07082-w

**Published:** 2023-01-19

**Authors:** Mengyang Wang, Rui Xia, Jiao Shi, Chunhua Yang, Yongqing Zhang, Zhengxian Xu, Cancan Yu, Ziyi Wu, Min Wang, Shangjie Chen, Hongdang Qu

**Affiliations:** 1 The First Affiliated Hospital of Bengbu Medical College, Bengbu, China; 2 Second Affiliated Hospital of Shenzhen University, Shenzhen, China

**Keywords:** rTMS, CPPS, Randomized controlled trial, RCT protocol

## Abstract

**Introduction:**

Nearly one in seven women worldwide suffers from chronic pelvic pain syndrome (CPPS) each year. Often, CPPS necessitates a combination of treatments. Studies have shown the good therapeutic effects of repetitive transcranial magnetic stimulation (rTMS) upon CPPS. We wish to undertake a randomized controlled trial (RCT) to observe the effect of high-frequency rTMS at different intensities upon CPPS.

**Methods and analyses:**

In this prospective, double-blinded RCT, 63 female CPPS participants will be recruited and randomized (1:1:1) to high-intensity rTMS, low-intensity rTMS, or sham rTMS. The control group will receive a 10-day course of conventional pelvic floor (PF) rehabilitation (neuromuscular stimulation, magnetic therapy, or light therapy of the PF). On the basis of conventional treatment, participants in the high-intensity rTMS group will receive pulses of 10 Hz with a resting motor threshold (RMT) of 110% for a total of 15,000 pulses. Participants in the low-intensity rTMS group will receive pulses of 10 Hz with an RMT of 80% with 15,000 pulses. The sham rTMS group will be subjected to sham stimulation with the same sound as produced by the real magnetic stimulation coil. The primary outcome will be determined using a visual analog scale, the Genitourinary Pain Index, Zung Self-Rating Anxiety Scale, and Zung Self-Rating Depression Scale. The secondary outcome will be determined by electromyography of the surface of PF muscles at baseline and after treatment completion.

**Ethics and dissemination:**

This study is approved by the Ethics Committee of Bao’an People’s Hospital, Shenzhen, Guangdong Province (approval number: BYL20211203). The results will be submitted for publication in peer-reviewed journals and disseminated at scientific conferences (Protocol version 1.0-20220709).

**Trial registration:**

Chictr.org.cn, ID: ChiCTR2200055615. Registered on 14 January 2022, http://www.chictr.org.cn/showproj.aspx?proj=146720. Protocol version 1.0-20220709.

## Strengths and limitations of this RCT design

- This is the first randomized controlled trial (RCT) focusing on the efficacy and acceptability of different intensities of repetitive transcranial magnetic stimulation (rTMS) for chronic pelvic pain syndrome (CPPS).
- Participants will be patients who come to Bao’an People’s Hospital needing pelvic floor (PF) rehabilitation and who have agreed to treatment of gynecological or urological diseases. Also, Bao’an People’s Hospital must have the relevant equipment.
- Treatment will be implemented and followed up by an experienced therapist. Sixty-three female participants with CPPS will be enrolled in this prospective, assessor-blind, three-arm RCT. They will be assigned (1:1:1) randomly to a high-intensity rTMS group, low-intensity rTMS group, or conventional-treatment group. This scheme can truly reflect the intervention effect of different intensities of rTMS upon CPPS.
- Electromyography of the surface of PF muscle will be undertaken before and after the intervention. We aim to investigate the effect of rTMS on the neuromuscular bioelectrical activity of PF muscles after treatment of CPPS.
- A methodological challenge will arise from blinded implementation and quality control of the intervention. The therapist and physician cannot act in a blinded manner. To minimize a performance bias, therapists and physicians will not be involved in recruitment or data analyses.

## Background

Since the 1990s, pelvic floor (PF) dysfunction has become a major problem affecting human health. In females, PF ligaments provide cord-like suspension and support for the stability of the uterus, ovaries, and vesicorectal urethra. PF muscles support internal organs like “bridges.” Therefore, the entire abdominopelvic region may be affected if disease occurs [1–3]. With women taking employment in all walks of life, the requirements for PF support are increasing, and mechanical imbalance in the pelvis and various triggers can promote the occurrence and development of various diseases.

Chronic pelvic pain syndrome (CPPS) can be defined as idiopathic pain in the lower abdomen that persists or is intermittent for >6 months and which causes stress to a woman’s psychology, life, and work [4]. Surveys have suggested only 2–10% of those who can receive efficacious treatment receive the diagnosis in the outpatient setting [5]. The impact of CPPS on women is reflected in emotional anxiety and depression in addition to pain, a decrease in quality of life (QoL), and abnormal aspects of physical sensitivity.

CPPS manifests as several peripheral symptoms, and studies have suggested that chronic pain involves systemic dysfunction in pain systems [6]. Patients with CPPS have a reduced pain threshold. Long-term pain leads to continuous transmission of pain-sensitizing inhibitory substances (e.g., glutamate) through nerve bundles in the spinal cord. This action increases the level of such substances in cerebrospinal fluid but also in key pain-processing areas, whereas the level of excitatory analgesic substances (e.g., opioid peptides) decreases [7]. All of these actions affect the occurrence and development of central sensitization and are the result of physiological plasticity and persistent changes in the central nervous system (CNS) [8, 9].

The CNS regulates the excitation and inhibition of nerves. The prefrontal cortex is a key region involved in the regulation of slow-acting pain and cognitive processing [10, 11]. Studies have shown the mechanism by which central regulation is feasible [12]. Imaging findings have demonstrated that the long-term effects of chronic pain can cause changes in gray matter density in the primary somatosensory cortex, hippocampus, and left amygdala [13].

Even these changes are related to not only pain production, but the occurrence of depression is significantly correlated with right hippocampal weight, but also the duration of pain; for example, symptom duration of less than 7.5 years was significantly related to the left amygdala [14, 15]. After receiving repetitive transcranial magnetic stimulation (rTMS), the increase in motor cortex (M1) excitability also elicits a potent analgesic effect [16]. This action can increase the concentrations of glutamate and glutamine, and biochemical tests have demonstrated glutamate in the prefrontal cortex of women suffering from CPPS [17, 18]. A change in the glutamine concentration has a negative correlation with the pain threshold [19]. Also, M1 is related to the pain level of healthy people and patients suffering from pelvic pain. M1 has a positive correlation with the pain threshold, which may be because M1 is involved mostly in pain loops [20, 21].

Therefore, in treatment programs used commonly for CPPS patients (e.g., myofascial manipulation, medium/low-frequency electrical stimulation, aerobic exercise, and traditional Chinese medicine) [22, 23], the treatment concept of central regulation from cortical targets is a rational approach [24, 25]. Also, rTMS (i) stimulates the brain area within 2 cm of the scalp, (ii) can trigger descending inhibitory neural pathways to act at the level of the dorsal horn to reduce chronic pain, (iii) can change neuronal activity, and (iv) affects the cerebral cortex associated with pain perception (e.g., prefrontal cortex and primary somatosensory cortex) [26, 27].

The allodynia and hyperalgesia elicited in the cortical projection pain areas of the pelvis in CPPS patients suggest that dysfunction of the central pain system may be very important in CPPS [6, 28]. Paresthesia and pain occur due to PF dysfunction. As the pain persists, systemic changes in the CNS occur, and the CNS remains in a state of high activity, showing disruption of pain regulation and enhanced central sensitization processes.

rTMS is considered to be a non-invasive method of brain stimulation [29]. rTMS has been shown to regulate the central lesions caused by chronic diseases by inhibiting and reversing neuronal sensitization [20, 30, 31]. Treatment guidelines based on TMS studies have suggested that the left prefrontal cortex could be a target for experimental application of chronic pain. rTMS was first used to treat pelvic and perineal pain in 2012 [32]. Louppe and colleagues carried out rTMS for 4 weeks with a high-frequency resting motor threshold (RMT) of 100% in two female patients (average pain duration >7.5 years) by stimulating M1. These two patients had continuous improvement in pain and QoL during 3 weeks of treatment and follow-up. In subsequent studies, high-frequency stimulation with an RMT of 110% [33] and 80% [34] was used as a more reliable option for treatment. In mechanistic studies, different intensities were considered to bring different outcomes, but the effect size was not uniformly positively correlated with intensity. In studies using a RMT of 80% and 110%, a RMT of 80% stimulated improvement of disease symptoms, but a reduction in pain was not obvious. Those studies were also influenced by non-consistent results caused by different disease subtypes and interventions. Therefore, design of a randomized controlled trial (RCT) to ascertain if treatment at different intensities of rTMS will have different effects on CPPS development is warranted.

We hypothesized that the treatment effect of rTMS upon CPPS is different at different intensities. We suggest that the effect in a low-intensity group will be different compared with that elicited by conventional treatment. In this way, we could determine the efficacy and safety of rTMS in CPPS treatment.

## Study design and methods

### Study design

**Fig. 1:**
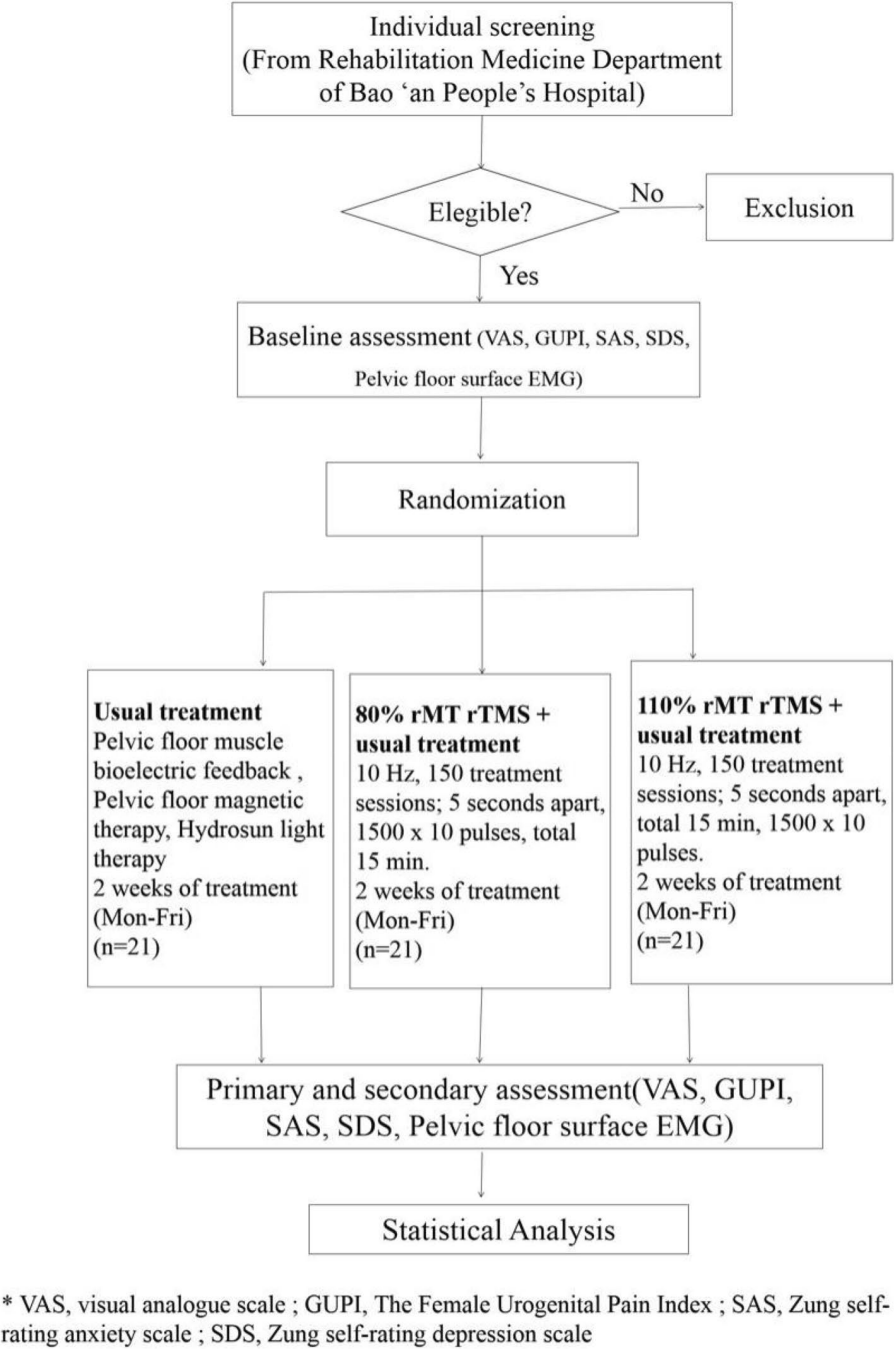
Flowchart of the RCT

Our study will be a triple-arm RCT with a 1:1:1 allocation ratio with allocation concealment and assessor blinding. A design framework of three groups in a RCT can minimize (as much as possible) the interference of other factors in the comparison between groups. Simultaneously, multiple comparisons can be made to ascertain the differences between the stimulated group and non-stimulated group, as well as between groups with different levels of stimulation. Sixty-three eligible participants will be randomly assigned to 10 high-intensity or low-intensity TMS (15 min each, five times a week) or sham stimulation groups, all receiving these stimuli in addition to their usual pelvic disease treatments. Results for pain and other symptoms will be assessed at baseline and 2 weeks (at the end of the intervention). Data collectors will be blinded to group assignments. The study procedure and schedule for outcome assessment for our RCT are presented in Fig. 1 and Table 1.

**Table 1:**
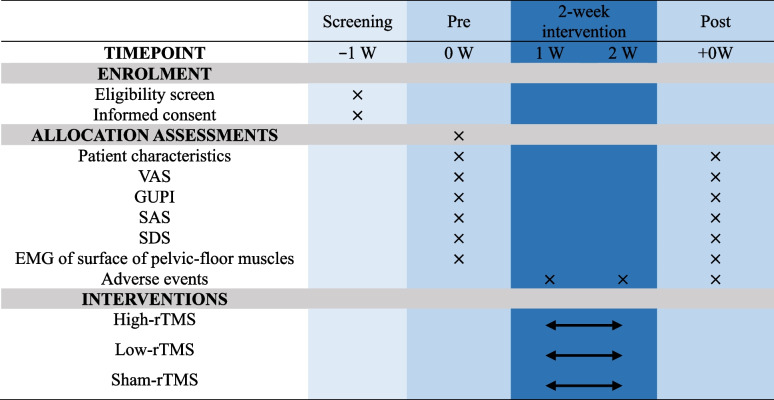
Schedule of enrollment, assessments, and interventions in the RCT

“×” indicates at which point of the RCT the respective assessments will take place

### Study population

The study population will be women between 20 and 60 years of age suffering from CPPS.

### Study setting

Researchers will be recruited from Bao’an People’s Hospital, a class III, class A university, affiliated community hospital in Shenzhen, China. Treatment will take place in the Department of Rehabilitation Medicine.

### Diagnostic criteria

According to European Association of Urology (EAU) Guidelines on Chronic Pelvic Pain (2019 version) and EUA Guidelines on Chronic Pelvic Pain (2019 updated version), participants with CPPS should have chronic pelvic pain: (i) with no proven infection or other obvious local disease that can explain the pain; (ii) associated with negative cognitive, behavioral, sexual, or emotional consequences; and (iii) with symptoms suggestive of lower urinary, sexual, intestinal, or gynecological dysfunction.

### Inclusion criteria

Eligible participants must meet the following criteria: (i) the diagnostic criteria and classification for CPPS in the EUA Guidelines on Chronic Pelvic Pain (2019 updated version) [4] must be met; (ii) aged 20–60 years with a history of CPPS; (iii) gynecological examination and auxiliary examination do not reveal clear pathologic changes; (iv) written informed consent will be obtained and subjects will voluntarily participate in randomized controlled trials; (v) no treatment within 3 months before RCT initiation; and (vi) conforming to the habit of right-handed hands.

### Exclusion criteria

Patients who have absolute and relative contraindications for rTMS will be excluded according to evidence-based guidelines for the use of rTMS therapy. The exclusion criteria will be patients (1) with a contraindication to TMS due to acute systemic or intracranial hemorrhagic diseases; (2) with cardiac metal membrane or a cardiac pacemaker; (3) with a tendency to bleed; (4) with severe heart disease, severe hypertension, or severe dysfunction of the heart, liver, lungs, or kidneys; (5) who use implantable electronic devices; (6) with intracranial infection/tumor; (7) with intracranial vascular metal stent implantation; (8) with fever; (9) for whom data on vital signs are not available; (10) with adverse reactions to magnetic therapy; (11) with open wounds or infections at the treatment site; (12) with malignant effusions, active pulmonary tuberculosis, or cancer; (13) with severe mental illness or epilepsy; and (14) who are pregnant.

### Withdrawal criteria and management

Participants will be allowed or required to withdraw from the RCT if they (i) are determined after evaluation to be unable to continue treatment due to adverse reactions or changes in condition; (ii) do not follow the treatment plan strictly or take other therapies while participating in the RCT; (iii) have significant changes in blood pressure, heart rate, or respiration during treatment; (iv) wish to leave the RCT; and (v) lost visitors.

### Recruitment

Recruitment will be conducted in the Rehabilitation Department of Shenzhen Bao’an People’s Hospital through online and offline methods such as networking, outpatient departments, and screening of inpatient medical records. Patients whose condition may worsen will be evaluated and screened to determine eligibility for participation based on the inclusion and exclusion criteria stated above. Inpatients wishing to participate will obtain permission from a physician, and a research assistant will obtain written informed consent from the participation before a therapeutic intervention.

### Randomization, allocation concealment, and blinding

After assessment at baseline, eligible participants will be assigned randomly to high-intensity stimulation, low-intensity stimulation, or conventional treatment. Randomly assigned sequences will be generated using the “PLAN” program of SAS 9.2 (Cary, NC, USA). Random serial numbers will be generated and placed in opaque, sealed envelopes. They will be administered independently by statisticians not involved in the recruitment, evaluation, or engagement of participants. Eligible participants will be notified of the assignment results by an independent research assistant over the telephone. We cannot allow therapists, or attending physicians, to be blinded to assigned treatment, but participants, outcome assessors, and statisticians will be blinded to group assignments.

### Unblinding

If there was a situation such as a medical need or emergency which required unblinding, the principal investigator will be informed and make the final decision.

### Intervention

Individuals will be assigned to a family physician at the intervention center. RCT compliance can be obtained daily through treatment records in the treatment instrument. Treatment will be recorded in the electronic medical record of participants. Relevant concomitant care and interventions that are permitted during the RCT will be shown to each participant.

All three groups will receive routine radiotherapy floor rehabilitation, including neuromuscular stimulation, magnetic therapy, or PF phototherapy. The instrument which will be used to administer neuromuscular stimulation of PF muscles will be Phenix-USB4 (Vivaltis, Paris, France). It uses 10-min low-frequency electrical stimulation and 15-min exercises for PF muscles for a total of 25 min per day. Magnetic therapy of the PF will be done using a Magneuro 60F system (Vishee Medical Technology, Nanjing, China). Moderate analgesic high-frequency stimulation for 20 min per day will be carried out. Light therapy will be undertaken using Hydrosun® Irradiator 500 (Hydrosun Medizintechnik, Müllheim, Germany). This employs direct irradiation of the pain site at a distance of 20–30 cm for 20 min per day. Conventional treatments will be carried out five times per week.

### Sham rTMS group

The sham group will receive rTMS with no actual output, stimulated with a fake magnetic stimulation coil that produces the same sound as the real stimulation coil, but without the magnetic field, and has induced a non-stimulating effect.

### High-intensity rTMS group

The high-intensity rTMS group will receive high-intensity rTMS in addition to conventional treatment. rTMS treatment will be administered using the CCY 1 stimulator (Yiruide, Wuhan, China). An “8”-shaped coil will focus the stimulation. The maximum output will be 2.2 T. The coil will be located in the center of the left M1, placed at 45° inclination from the scalp. The output intensity will be a RMT of 110%. A total of 1500 pulses will be administered at each treatment (10 pulses delivered in each sequence, repeat 150 times, 5 s apart). rTMS will be given at 10 Hz for 15 min, and the stimulation site will be M1. rTMS will be administered once a day, five times a week for 2 weeks. Ten sessions, for a total of 15,000 stimulations, will be administered.

### Low-intensity rTMS group

Patients in the low-intensity rTMS group will use identical equipment to those in the high-intensity group. However, the stimulation intensity will be a RMT of 80%. The number of pulses, interval between pulses, and stimulation frequency will be identical to those in the high-intensity group.

### Outcome assessment

The variables to be assessed will be patient characteristics, primary outcome, and secondary outcome. Patient characteristics at baseline will be documented. Primary and secondary outcomes will be measured at baseline and at the end of the intervention. The time points of result recording will be denoted as T0 and T10. Primary and secondary outcomes will be evaluated by experienced staff at Shenzhen Bao’an People’s Hospital, who will not be aware of the results of participant allocation.

#### Patient characteristics

Recruiters will use a self-designed questionnaire to collect demographic characteristics (e.g., sex, age, height, weight, and reproductive status) as well as a history of disease or drug use. Scores for a visual analog scale (VAS), the Genitourinary Pain Index (GUPI), Zung Self-Rating Anxiety Scale (SAS), and Zung Self-Rating Depression Scale (SDS) will be calculated. Measurements at baseline will be completed before the first treatment.

#### Primary outcome

Pain severity will be based on the VAS score [35]. A VAS is a 100-mm line where “zero” indicates complete painlessness and “10” indicates the most severe pain imaginable. The patient marks pain severity on this line. The simplicity of a VAS enables its use for pain assessment. Changes before and after treatment were compared by the decrease in VAS.

### Secondary outcomes

GUPI [36] will be employed to assess the severity of symptoms in women with urogenital pain. GUPI is a modification of the National Institutes of Health–Chronic Prostatitis Symptom Index and includes female-specific pain (pain at the vaginal entrance, urethral pain, pain during/after sexual intercourse). There are 10 pain items with a total pain subscale score of 0–13, two urinary symptom items with a total urine subscale score of 0–10, and three QoL items with a total QoL subscale score of 0–12.

SAS [37, 38] is a norm-referenced scale which enjoys widespread use as a screening device for anxiety disorders. The scale measures mood over the previous week with a score that is multiplied by 1.25. An SAS score of <50 suggests a “normal” mood, that of 50–59 indicates “mild depression,” that of 60–69 denotes “moderate depression,” and that of ≥70 indicates “major depression.” Studies [6, 39] have suggested that the mood changes of patients with long-term pain are obvious and that a self-rating scale can be used to distinguish their emotions in a preliminary manner.

SDS [40] consists of 20 items divided into four self-rating scales. SDS is used to evaluate the subjective feelings of anxious patients within 2 weeks. The score is multiplied by 1.25. A final SDS score of <50 denotes a “normal” mood, that of 50–59 indicates “mild depression,” that of 60–69 denotes “moderate depression,” and that of ≥70 indicates “major depression” [41].

Data for electromyography at the PF surface will be collected at baseline and after the intervention. Electromyography will be conducted in the Rehabilitation Room of Bao’an People’s Hospital. Participants will be assessed based on the Glazer procedure using a myoelectric PF biofeedback instrument (Medlander Technology, Nanjing, China). The data collected will be as follows: pre-resting phase, variogram 1, rapid-contraction phase, rise time, recovery time, slow-muscle phase, variogram 2l endurance-test phase, variogram 3, post-first 10-s ratio, post-resting phase, and variogram 4 [42–44].

### Safety and participant compensation

During the intervention, participants will be monitored for adverse events. Participants will be asked about adverse events after each treatment until the adverse event is resolved. If headache occurs after treatment, relaxation therapy will be given. Or other conditions such as a fall during the treatment will be treated free of charge by a professional therapist until recovery. A participant who is found to be at risk (e.g., suicide and neglect) to him/herself or others, or who has a serious adverse event, will be referred to the relevant clinical services. Causality will be assessed in relation to rTMS and the severity of the adverse event. Serious adverse events will be reported to the Ethics Committee of Bao’an People’s Hospital within 72 h.

### Sample size

According to the study by Pinot-Monange and colleagues [33], the effect size was 0.49 using *β* = 0.15 and *α* = 0.05. Pain scores will be taken as the main measurement standard, and G*Power 1.9.2 (www.psychologie.hhu.de/) will be employed. The study cohort was calculated to be ~51 patients. Taking into account a dropout of 20%, each group will include 21 people from each group. Sixty-three participants will be required for this RCT.

### Statistical analyses

SPSS 26 (IBM, Armonk, NY, USA) will be employed to analyze data. Statistical analyses will be conducted by independent statisticians. First, the Kolmogorov–Smirnov test will be used to ascertain if a normal distribution of data is present. If the data have a normal distribution, then analysis of variance with repeated measures will be used to compare the mean pain scores for each group. *P* < 0.05 will be considered significant. Continuous variables in the measures of primary and secondary outcomes will be expressed as the mean and standard deviation (normal distribution) or median value (non-normal distribution). Categorical variables will be described as frequencies or percentages. Subgroup analysis is not applicable in this RCT. Missing data (loss to follow-up, death, withdrawal, etc.) will be updated using the multiple-imputation approach.

The significance of adverse reactions will be analyzed using the chi-square test or Fisher’s exact test. If statistical analyses between groups are not possible due to insufficient power, adverse events will be tabulated and summarized using descriptive statistics.

### Collection and management of data

Data will be collected by an outcome evaluator using a case report form. The letter will be transcribed into a password-protected document by a research assistant until being “unlocked” at the end of the study. Additionally, missing data (loss to follow-up, death, withdrawal, etc.) will be updated using the multiple-imputation approach.

### Oversight and monitoring

The principal investigator and other co-investigators are doctors and professors of rehabilitation who will be responsible for overseeing and monitoring the entire trial. Subject diagnosis, recruitment, and informed consent signing will be handled by an independent hospital-affiliated rehabilitation physician. The progress, adverse events, and data quality of the trial will be evaluated by an independent data testing committee composed of Bao’an People’s Hospital Ethics Committee members and independent therapists who are not affiliated with the study team or sponsors. The study team will meet monthly with the data testing committee to monitor the progress and quality of the study. There will be no interim analysis. Statistical analysis is conducted only after the trial, and if individual participants report serious adverse effects or deterioration during the course of the trial, the principal investigator will make a decision to terminate the trial.

### Involvement of patients and the public

Before recruitment, individual patients and experienced therapists not associated with recruitment will be invited to investigate the RCT design and feasibility of the intervention. Based on their comments, the evaluation method and timing of treatment will be adjusted to avoid interference with routine treatment arrangements. Patients or members of the public will not be involved in recruitment or data implementation and measurement. Results will be sent to participants via written reports and email. The burden of the interventions included in this RCT will not be assessed by patients. During the intervention, participants were contracted to a family physician at the hospital (including records of all visits and regular follow-up visits). The data of the participants in the intervention interruption or the intervention program are retained, and the reasonable and exposed data can be excluded and counted according to the needs of the participants.

### Ethical approval and communication

The protocol for this RCT was approved (BYL-20211203) by the Ethics Committee of Shenzhen Bao’an People’s Hospital in December 2021. Changes to the protocol will be submitted to this Ethics Committee for approval. This RCT was registered on 14 January 2022 at www.ChiCTR.org and can be identified as ChiCTR2200055615. Written informed consent of all participants will be obtained after they have read an information manual describing the project and potential risks and benefits and if they have questions to discuss with the investigator. Collected digital data will be stored in password-protected files. Only the data analyst will have access to the data at the end of the RCT. RCT results will be published in a peer-reviewed journal following standards set by the International Board of Medical Journal Editors and will not involve professional authors, with undetermined data being available in a public repository at the time of publication. RCT results will also be communicated through scientific meetings.

## Discussion

As a non-invasive method of brain stimulation, rTMS has a deeper role than other methods used to study peripheral regulation [17, 31, 45, 46]. Several studies have revealed that high-frequency rTMS in M1 has therapeutic value for chronic intractable pain and generalized pain and compared it with other methods of central regulation. rTMS has been supported by more RCT data and has a higher level of evidence than that of other methods [39]. An analgesic effect can be maintained for 2–3 days and up to 8 days after rTMS of M1 [47]. High-frequency rTMS of M1 appears to be suitable for middle-aged women with recurrent idiopathic pain attacks.

Our RCT has been designed to compare rTMS of different intensities. Thordstein and colleagues [48] demonstrated that the central region affected by rTMS may elicit no response at high or low levels of intensity but that an increase in stimulus of just 10% may produce significant changes. For diseases such as epilepsy [48–50], schizophrenia, and cognitive impairment, experimental studies have suggested that different intensities of rTMS affect astrocytes and cerebral blood flow. In studies investigating use of rTMS for CPPS or other types of chronic pain, uniform parameters (frequency, intensity, duration) or type of stimulation (transcranial magnetic coil) has not been used. In some sessions with an RMT of 80%, the improvement in QoL, anxiety, and depression was not significant. Therefore, we believe in the necessity of designing a RCT to investigate if there is a difference in CPPS if high-frequency rTMS at different intensities is employed.

The emotional and psychological problems of CPPS cannot be ignored [6, 51, 52]. Chronic pain causes abnormal changes in the strength of PF muscles but also affects intestinal activity, urinary system, somatic psychology, QoL, anxiety, and depression. Therefore, we consider it necessary to include pain, urinary outcome, anxiety, depression, and surface electromyography in outcome assessments.

In this study, the age of the subjects was considered to be over 20 years old, which may affect the validity of the experiment. However, we referred to the age of marriage and childbearing that met the criteria. In addition, the factors such as region and race may also have an influence, so this single-center experiment needs to be improved.

## Conclusions

This will be the first RCT to evaluate the impact of high-frequency rTMS at different intensities upon CPPS. It may provide a reference for future clinical treatment and research of CPPS.

## Data Availability

Not applicable. No data have been generated. Raw data sharing will be available after 31 December 2023 by contacting the author of this article.

## References

[1] Ashton-Miller JA, Delancey JO (2007). Functional anatomy of the female pelvic floor. Ann N Y Acad Sci.

[2] Easley DC, Abramowitch SD, Moalli PA (2017). Female pelvic floor biomechanics: bridging the gap. Curr Opin Urol.

[3] Xu W, Wang H, Jiang Y (2021). Effects of pelvic biomechanical changes on female pelvic floor dysfunction. J Med Biomech.

[4] Guidelines E (2019). EAU guidelines on chronic pelvic pain.

[5] Till SR, As-Sanie S, Schrepf A (2019). Psychology of chronic pelvic pain: prevalence, neurobiological vulnerabilities, and treatment. Clin Obstet Gynecol.

[6] Grinberg K, Sela Y, Nissanholtz-Gannot R (2020). New insights about chronic pelvic pain syndrome (CPPS). Int J Environ Res Public Health.

[7] Yang S, Chang MC (2019). Chronic pain: structural and functional changes in brain structures and associated negative affective states. Int J Mol Sci.

[8] Sluka KA, Clauw DJ (2016). Neurobiology of fibromyalgia and chronic widespread pain. Neuroscience.

[9] Kuner R (2010). Central mechanisms of pathological pain. Nat Med.

[10] Thompson JM, Neugebauer V (2019). Cortico-limbic pain mechanisms. Neurosci Lett.

[11] Kang D, McAuley JH, Kassem MS (2019). What does the grey matter decrease in the medial prefrontal cortex reflect in people with chronic pain?. Eur J Pain.

[12] Yang S, Chang MC (2020). Effect of repetitive transcranial magnetic stimulation on pain management: a systematic narrative review. Front Neurol.

[13] Huang X, Chen J, Liu S (2021). Impaired frontal-parietal control network in chronic prostatitis/chronic pelvic pain syndrome revealed by graph theoretical analysis: a DTI study. Eur J Neurosci.

[14] Baheti AD, Nicola R, Bennett GL, et al. Magnetic resonance imaging of abdominal and pelvic pain in the pregnant patient. Magn Reson Imaging Clin N Am. 2016;24(2):403–17. https://pubmed.ncbi.nlm.nih.gov/27150326/. 10.1016/j.mric.2015.11.007 27150326

[15] Morotti M, Vincent K, Becker CM (2017). Mechanisms of pain in endometriosis. Eur J Obstet Gynecol Reprod Biol.

[16] Attal N, Poindessous-Jazat F, De Chauvigny E (2021). Repetitive transcranial magnetic stimulation for neuropathic pain: a randomized multicentre sham-controlled trial. Brain.

[17] Lefaucheur JP, Aleman A, Baeken C (2020). Evidence-based guidelines on the therapeutic use of repetitive transcranial magnetic stimulation (rTMS): an update (2014-2018). Clin Neurophysiol.

[18] Che X, Cash RFH, Luo X (2021). High-frequency rTMS over the dorsolateral prefrontal cortex on chronic and provoked pain: a systematic review and meta-analysis. Brain Stimul.

[19] Simis M, Reidler JS, Duarte Macea D (2015). Investigation of central nervous system dysfunction in chronic pelvic pain using magnetic resonance spectroscopy and noninvasive brain stimulation. Pain Pract.

[20] Nardone R, Versace V, Sebastianelli L (2019). Transcranial magnetic stimulation and bladder function: a systematic review. Clin Neurophysiol.

[21] Giannoni-Luza S, Pacheco-Barrios K, Cardenas-Rojas A (2020). Noninvasive motor cortex stimulation effects on quantitative sensory testing in healthy and chronic pain subjects: a systematic review and meta-analysis. Pain.

[22] Cottrell AM, Schneider MP, Goonewardene S (2020). Benefits and harms of electrical neuromodulation for chronic pelvic pain: a systematic review. Eur Urol Focus.

[23] Carey ET, Moore K (2019). Updates in the approach to chronic pelvic pain: what the treating gynecologist should know. Clin Obstet Gynecol.

[24] Perry CP (2001). Current concepts of pelvic congestion and chronic pelvic pain. Jsls.

[25] Wozniak S (2016). Chronic pelvic pain. Ann Agric Environ Med.

[26] Leung A, Donohue M, Xu R (2009). rTMS for suppressing neuropathic pain: a meta-analysis. J Pain.

[27] Garland EL (2012). Pain processing in the human nervous system: a selective review of nociceptive and biobehavioral pathways. Prim Care.

[28] Kaya S, Hermans L, Willems T (2013). Central sensitization in urogynecological chronic pelvic pain: a systematic literature review. Pain Physician.

[29] Rossi S, Antal A, Bestmann S (2021). Safety and recommendations for TMS use in healthy subjects and patient populations, with updates on training, ethical and regulatory issues: expert guidelines. Clin Neurophysiol.

[30] Zhang KL, Yuan H, Wu FF (2021). Analgesic effect of noninvasive brain stimulation for neuropathic pain patients: a systematic review. Pain Ther.

[31] O’Connell NE, Marston L, Spencer S (2018). Non-invasive brain stimulation techniques for chronic pain. Cochrane Database Syst Rev.

[32] Louppe JM, Nguyen JP, Robert R (2013). Motor cortex stimulation in refractory pelvic and perineal pain: report of two successful cases. Neurourol Urodyn.

[33] Pinot-Monange A, Moisset X, Chauvet P (2019). Repetitive transcranial magnetic stimulation therapy (rTMS) for endometriosis patients with refractory pelvic chronic pain: a pilot study. J Clin Med.

[34] Cervigni M, Onesti E, Ceccanti M (2018). Repetitive transcranial magnetic stimulation for chronic neuropathic pain in patients with bladder pain syndrome/interstitial cystitis. Neurourol Urodyn.

[35] Senechal Q, Echegut P, Bravetti M (2021). Endovascular treatment of pelvic congestion syndrome: visual analog scale follow-up. Front Cardiovasc Med.

[36] Clemens JQ, Calhoun EA, Litwin MS (2009). Validation of a modified National Institutes of Health chronic prostatitis symptom index to assess genitourinary pain in both men and women. Urology.

[37] Dunstan DA, Scott N (2020). Norms for Zung’s self-rating anxiety scale. BMC Psychiatry.

[38] Zung WW (1971). A rating instrument for anxiety disorders. Psychosomatics.

[39] Jin Y, Xing G, Li G (2015). High frequency repetitive transcranial magnetic stimulation therapy for chronic neuropathic pain: a meta-analysis. Pain Physician.

[40] Zung WW (1965). A self-rating depression scale. Arch Gen Psychiatry.

[41] Grandi G, Xholli A, Ferrari S (2013). Intermenstrual pelvic pain, quality of life and mood. Gynecol Obstet Invest.

[42] Rocca Rossetti S (2016). Functional anatomy of pelvic floor. Arch Ital Urol Androl.

[43] Hagen S, Elders A, Stratton S (2020). Effectiveness of pelvic floor muscle training with and without electromyographic biofeedback for urinary incontinence in women: multicentre randomised controlled trial. BMJ.

[44] Chmielewska D, Stania M, Kucab-Klich K (2019). Electromyographic characteristics of pelvic floor muscles in women with stress urinary incontinence following sEMG-assisted biofeedback training and Pilates exercises. PLoS One.

[45] Moisset X, Lanteri-Minet M, Fontaine D (2020). Neurostimulation methods in the treatment of chronic pain. J Neural Transm (Vienna).

[46] Knotkova H, Hamani C, Sivanesan E (2021). Neuromodulation for chronic pain. Lancet.

[47] Lefaucheur JP, Drouot X, Nguyen JP (2001). Interventional neurophysiology for pain control: duration of pain relief following repetitive transcranial magnetic stimulation of the motor cortex. Neurophysiol Clin.

[48] Thordstein M, Saar K, Pegenius G (2013). Individual effects of varying stimulation intensity and response criteria on area of activation for different muscles in humans. A study using navigated transcranial magnetic stimulation. Brain Stimul.

[49] Wang J, Huang L, Wei L (2021). Factors affecting the efficacy of repetitive transcranial magnetic stimulation for patients with Alzheimer’s disease. Zhejiang Da Xue Xue Bao Yi Xue Ban.

[50] Padberg F, Zwanzger P, Keck ME (2002). Repetitive transcranial magnetic stimulation (rTMS) in major depression: relation between efficacy and stimulation intensity. Neuropsychopharmacology.

[51] Passavanti MB, Pota V, Sansone P (2017). Chronic pelvic pain: assessment, evaluation, and objectivation. Pain Res Treat.

[52] Clemens JQ, Mullins C, Kusek JW (2014). The MAPP research network: a novel study of urologic chronic pelvic pain syndromes. BMC Urol.

